# Economic Burden of Late-Stage Age-Related Macular Degeneration in Bulgaria, Germany, and the US

**DOI:** 10.1001/jamaophthalmol.2024.4401

**Published:** 2024-10-31

**Authors:** Nabin Paudel, Laura Brady, Petia Stratieva, Orla Galvin, Beverly Lui, Iris Van den Brande, Jean-Pierre Malkowski, Mayvis Rebeira, Simon MacAllister, Tara O’Riordan, Avril Daly

**Affiliations:** 1 Retina International, Dublin, Ireland; 2 Apellis International, Zug, Switzerland; 3 F. Hoffmann-La Roche, Basel, Switzerland; 4 Novartis Pharma, Basel, Switzerland; 5 Alexion Pharma Canada Corp, AstraZeneca Rare Disease, Mississauga, Ontario, Canada; 6 Ernst & Young Ireland, Dublin, Ireland; 7 FutureNeuro Science Foundation Ireland Research Centre, Royal College of Surgeons in Ireland University of Medicine and Health Sciences, Dublin, Ireland

## Abstract

**Importance:**

Age-related macular degeneration (AMD) is a highly prevalent and debilitating retinal condition that affects more than 200 million people globally, with the severe late-stage forms—neovascular AMD (nAMD) and geographic atrophy (GA)—affecting more than 11 million people globally. However, much is unknown about the economic burden of the disease.

**Objective:**

To estimate the economic burden associated with late-stage AMD in Bulgaria, Germany, and the US at the societal level.

**Design, Setting, and Participants:**

This study used the prevalence approach cost-of-illness economic modeling to estimate costs attributable to late-stage AMD. Data on health care resource utilization, well-being, and productivity were obtained via primary data collection. Additional data required for the model were sourced from available published literature. Data were collected from January 2021 to March 2022, and analyzed from April to July 2022. Participants older than 50 years residing in Bulgaria, Germany, and the US who were diagnosed with late-stage AMD (nAMD or any form of GA) in 1 or both eyes and caregivers who care for people diagnosed with late-stage AMD were recruited through ophthalmological clinics in Bulgaria and Germany and via online newsletters and social media in the US.

**Main Outcomes and Measures:**

The main outcomes were direct medical costs (disease-related health care expenditures), indirect medical costs (care support and assistive technology), well-being cost (loss of well-being), and productivity cost (loss in productivity due to the diseases for both patient and caregivers).

**Results:**

Of the 128 individuals with late-stage AMD in this study, 80 (62%) were female, and 120 (94%) were aged 60 years or older. Of the 61 caregivers, 43 (70%) were female and 55 (91%) were aged 45 years or older. Estimated per-annum total costs attributable to late-stage AMD were €449.5 million ($512.5 million) in Bulgaria, €7.6 billion ($8.6 billion) in Germany, and €43.2 billion ($49.4 billion) in the US. Across all countries, 10% to 13% of the total cost incurred was attributed to direct medical costs. In Germany and Bulgaria, the biggest contributor to the total economic burden was reduced well-being (67% and 76%, respectively), whereas in the US, loss of productivity (42%) was the biggest contributor.

**Conclusions and Relevance:**

The findings of this study indicate a substantial burden of late-stage AMD on patients and caregivers in the US, Germany, and Bulgaria. Across the 3 countries, reduced well-being and loss of productivity were relatively large contributors to the total economic burden. Implementing measures to reduce AMD incidence, delay disease progression, and alleviate humanistic burden may help reduce the economic burden of late-stage AMD.

## Key Points

**Question:**

What is the economic burden of late-stage age-related macular degeneration (AMD) in Bulgaria, Germany, and the US?

**Findings:**

In this economic evaluation study, the per-annum economic burden of late-stage AMD in the 3 studied countries was large, with reduced well-being and loss in productivity accounting for most of the cost burden across countries. Direct medical costs contributed to a relatively small portion of the total cost.

**Meaning:**

The findings suggest that the financial toll associated with late-stage AMD is substantial and warrants investments in AMD care and treatments.

## Teaser

This economic evaluation study estimates the economic burden of late-stage age-related macular degeneration in 3 countries.

## Introduction

Age-related macular degeneration (AMD) is a serious public health issue, representing the leading cause of visual impairment and blindness in high-income countries.^1^ Globally, approximately 8.7% of people older than 45 years have some form of AMD, affecting around 200 million individuals. Projections indicate that the number of individuals with any form of AMD will increase to 288 million by 2040.^2^ AMD is a progressive disease of the central retina leading to blurring, distortion, and loss of central vision. Major risk factors associated with AMD are increasing age, smoking, and diet.^3^ Late-stage AMD presents a higher risk of severe vision impairment compared to early and intermediate stages. The global prevalence of late-stage AMD is 0.37%, affecting around 12 million people, and it is projected that the number of people with the late-stage AMD will increase to 18 million by 2040.^2^ Late-stage AMD can be categorized into 2 types: atrophic (also referred to as geographic atrophy [GA]) and exudative (often referred to as neovascular AMD [nAMD] or wet AMD). GA is the more common form, with early and intermediate stages of AMD typically progressing slowly to the atrophic stage. In contrast, nAMD can develop rapidly from early or intermediate stages. While there are several treatment options available for nAMD, to date, there are 2 US Food and Drug Administration–approved therapies for GA, but neither has been approved by the European Medicines Agency. These therapies have shown anatomic benefits but not visual function improvements across prespecified secondary outcomes. Several other GA treatments are currently in development.^4,5^

In addition to the considerable deleterious effect AMD can have on several domains of visual function, such as visual acuity, visual field, color vision, and contrast sensitivity, and association with impaired patient-reported vision-related quality of life,^6^ AMD can be associated with adverse economic outcomes.^7,8,9,10^ Specifically, these outcomes can extend beyond the direct medical costs associated with treatment. People living with AMD often require additional support, including caregiving services, vision aids, and rehabilitation, further escalating the economic burden. The association of AMD with an individual’s and caregiver’s emotional well-being and productivity remains poorly understood.^11^ Several previous studies have reported the economic burden of AMD, but none, to our knowledge, has taken a more comprehensive approach to evaluate the disease burden for both people living with AMD and caregivers.^7,8,9,10,11,12^ Understanding the comprehensive economic burden of AMD could aid with decision-making in health care policy, economic planning, health technology assessments, medical research, and public health initiatives, potentially leading to better health outcomes and more efficient use of resources. Here, we endeavor to estimate the annual total burden of late-stage AMD, including nAMD and GA, in Bulgaria, Germany, and the US. We aim to evaluate the toll of late-stage AMD in countries with diverse health care systems, access hurdles, socioeconomic statuses, and cultural traits.

## Methods

### Data Collection

From January 2021 to March 2022, people aged 50 years and older with late-stage AMD (nAMD or any form of GA) in 1 or both eyes were surveyed, alongside caregivers. Participants were recruited through ophthalmological clinical practices in Bulgaria and Germany. In the US, an online survey was distributed via email newsletters and social media groups targeting patients with late-stage AMD and caregivers. Data were analyzed from April to July 2022. This study was approved by the Retina International Ethical Review Board and performed in accordance with the tenets of the Declaration of Helsinki. Written informed consent was obtained for all participants, and no incentive was provided for completing the survey. This study was conducted and reported in accordance with the Consolidated Health Economic Evaluation Reporting Standards (CHEERS) reporting guideline.^13^

### Study Approach

The study used the cost-of-illness prevalence approach to estimate costs attributable to late-stage AMD.^14^ The cost-of-illness prevalence approach was chosen due to its comprehensive ability to provide a snapshot of the current economic burden, aiding in immediate resource allocation and policy planning.^14^ Data informing the economic model and associated cost categories (Figure; eFigure 1 in Supplement 1) were gathered via a targeted literature review and complemented through primary data collection from patients and caregivers via surveys. Cost data for clinical examinations, prescriptions, and medical devices were obtained from public sources and clinician interviews. When public data were unavailable, subscription databases were used and validated with clinicians. Two cross-sectional surveys, one for patients and one for caregivers, were codesigned following consultation with an internationally representative group of patients and patient advocates (surveys included in Supplement 1). The patient survey covered questions in relation to health care utilization, self-reported vision health, emotional well-being, productivity, and association with activities of daily living. The caregiver survey assessed the association of caregiving responsibilities with productivity, emotional well-being, and activities of daily living.

**Figure.**
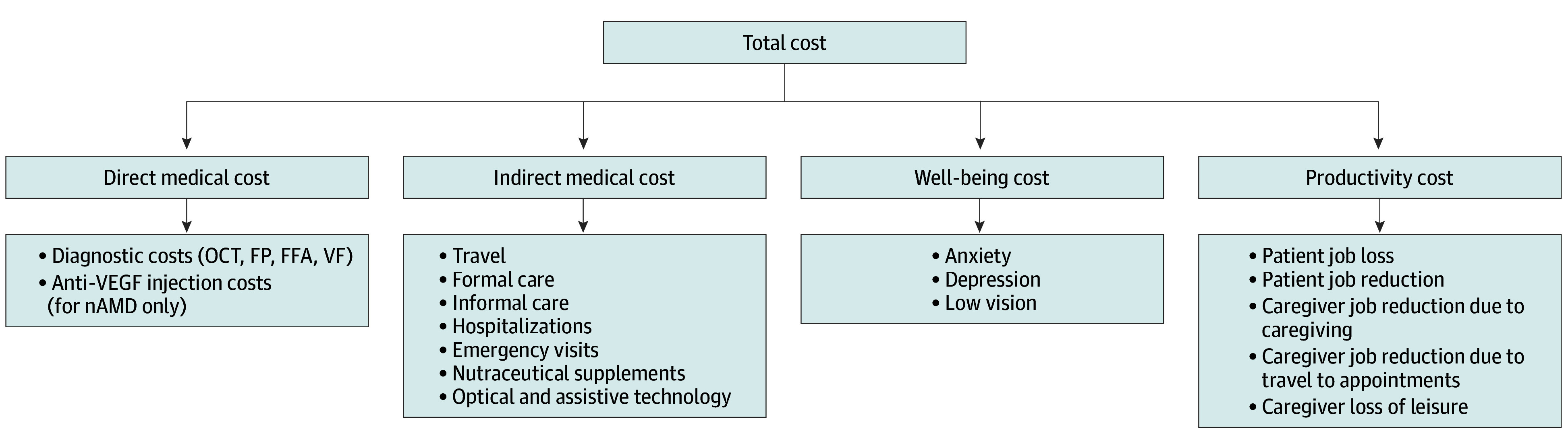
Cost of Illness Model Used for the Study FFA indicates fundus fluorescein angiography; FP, fundus photography; nAMD, neovascular age-related macular degeneration; OCT, optical coherence tomography; VEGF, vascular endothelial growth factor; VF, visual field.

### Prevalence of Late-Stage AMD

Published studies that reported the prevalence of neovascular, wet, nonexudative AMD; or advanced dry AMD; or GA were assessed to determine prevalence. Considerable variability in prevalence rates was observed; therefore, 3 prevalence scenarios have been considered: low, mid-, and high. The mid-prevalence rate represents the middle reported rate between the high and low rates. In the assessment of Bulgaria, 2 prevalence rate studies were sourced^15,16^; however, specific GA or nAMD data were lacking. Therefore, the rates for the US were assumed for Bulgaria, as the US rates were lower and hence a more conservative assumption. Three prevalence studies were identified for Germany^16,17,18^ and for the US.^19,20,21^

### Statistical Analysis

The costs associated with late-stage AMD were grouped into 4 main categories: direct medical costs, indirect medical costs, well-being costs, and productivity costs. Direct medical costs encompassed those related to diagnosis and treatment of the disease. Indirect medical costs captured costs associated with assistive technology, treatment of fall-related injuries (emergency visit or inpatient visits), formal care (home help or nursing home care), travel to and from eye care appointments, and nutraceutical supplements. Well-being costs were determined using a nonfinancial approach by monetizing the reported sequala (low vision, anxiety, and depression) of the disease. The cost of each sequalae was calculated by multiplying the proportion of individuals with late-stage AMD that reported being affected by the sequala, by the disability weight of the sequala, by the willingness to pay for a year of full health in each country.

Productivity costs accounted for the loss of productivity and employment changes for patients and caregivers. Parameters included patient job loss, patient job reduction, caregiver job reduction due to caregiving responsibilities, caregiver job reduction due to providing transport to eye health care appointments, and caregiver loss of leisure time due to providing transport.

Data collected for each category were then combined for each country to estimate the societal cost of late-stage AMD. For comparability, all figures in this report are provided in euros, with the 2020 average exchange rate used to convert from dollars and Bulgarian levs (Bulgarian levs converted to euros by multiplying by 0.511; US dollars to euros by 0.877). See eTables 1-8 and eFigures 1-5 in Supplement 1 regarding data sources and cost calculation methods. The total economic burden of late-stage AMD in each country was estimated by combining financial (direct medical, indirect medical, and productivity costs) and nonfinancial costs (well-being).

## Results

A total of 128 individuals diagnosed with late-stage AMD were included in the study (80 [62%] female and 48 [38%] male; 120 [94%] were aged 60 years or older) completed the survey and were included. Sixty-one caregivers (43 [70%] female and 17 [30%] male; 55 [91%] were aged 45 years or older) completed the survey and were included (Table 1).

**Table 1.** Age Distribution and Type of Age-Related Macular Degeneration (AMD) Among People Living With AMD and Caregivers

Variable	Individuals, No.		
	Bulgaria	Germany	US
People living with AMD			
Total	27	41	60
Geographic atrophy	17	10	30
Neovascular AMD	10	31	30
Age range, y			
55-59	0	1	4
60-64	0	3	4
65-69	4	9	9
70-74	6	7	19
75-79	4	8	12
80-84	8	12	8
85-89	3	1	3
90-94	2	0	1
Caregivers			
Total	23	20	18
Geographic atrophy	16	5	4
Neovascular AMD	7	15	14
Age range, y			
18-24	1	0	0
25-34	2	0	0
35-44	1	1	0
45-54	6	4	6
55-64	8	5	4
65-74	2	4	5
75-84	3	4	5

### Prevalence of Late-Stage AMD and Economic Burden

Midlevel prevalence rates of late-stage AMD in the studied countries ranged from 0.10 to 0.20 with an estimated impacted population between 11 420 and 490 440. Table 2 presents identified prevalence rates and estimated populations impacted by GA and nAMD. The total economic burden of late-stage AMD in the studied countries was substantial. Across all countries, only a fraction (10% to 13%) of the total cost incurred was attributed to direct medical costs. In Germany and Bulgaria, the biggest contributor to the total economic burden was reduced well-being (67% and 76%, respectively), whereas in the US, loss of productivity (42%) was the biggest contributor.

**Table 2.** Prevalence of Late-Stage Age-Related Macular Degeneration (AMD) and Estimated Affected Population in the Studied Countries in 2021

	Median (range)	
	Mid-prevalence rate (low-high)	Estimated impacted population in 2021
**Bulgaria**		
Geographic atrophy	0.10 (0.01-0.20)	11 420 (1683-24 339)
Neovascular AMD	0.10 (0.02-0.60)	11 420 (2584-55 858)
**Germany**		
Geographic atrophy	0.20 (0.02-0.40)	191 450 (25 657-414 482)
Neovascular AMD	0.20 (0.04-1.20)	191 450 (44 607-1 077 716)
**US**		
Geographic atrophy	0.10 (0.01-0.20)	490 440 (76 460-1 034 950)
Neovascular AMD	0.10 (0.02-0.60)	490 440 (108 902-2 170 420)

### Bulgaria

The economic burden of late-stage AMD was estimated to range from €84.6 million ($96.4 million) to €1.5 billion ($1.7 billion) in 2021. Considering the midvalue prevalence rate, the total cost attributable was estimated at €449.5 million ($512.5 million) (Table 3). In both GA and nAMD, the cost associated with well-being was the major contributor to the overall cost, accounting for 87% and 64% of the total cost, respectively. Financial costs incurred due to GA and nAMD were €27.45 million ($31.30 million) and €81.4 million ($92.8 million), respectively (Table 3). In GA, 97% of the total cost incurred was due to the loss of productivity in patients. In nAMD, 99% of the total cost was attributed to the loss of productivity in patients, with caregivers contributing the remaining 1% of the cost. Per-annum individual cost incurred due to GA and nAMD was estimated at €19 311 ($22 019) and €20 047 ($22 858), respectively (Table 4).

**Table 3.** Estimated Per-Annum Population Cost According to Age-Related Macular Degeneration (AMD) Type and Cost Categories Across the Studied Countries

	Cost (% of total cost), €^a^		
	Geographic atrophy	Neovascular AMD	All AMD
**Bulgaria**			
Direct medical cost	3.50 million (1.6)	40.15 million (17.5)	43.65 million (9.7)
Indirect medical cost	9.26 million (4.2)	23.92 million (10.4)	33.18 million (7.4)
Well-being cost	193.10 million (87.6)	147.57 million (64.4)	340.67 million (75.8)
Productivity cost	14.69 million (6.7)	17.31 million (7.6)	32.00 million (7.1)
Total	220.54 million (100)	228.94 million (100)	449.48 million (100)
Financial costs	27.45 million (12.4)	81.38 million (35.5)	108.83 million (24.2)
**Germany**			
Direct medical cost	34.02 million (0.9)	962.68 million (24.5)	996.68 million (13)
Indirect medical cost	143.87 million (3.8)	556.62 million (14.1)	700.49 million (9)
Well-being cost	3.24 billion (86.7)	1.88 billion (48)	5.12 billion (67)
Productivity cost	317.42 million (8.5)	524.11 million (13.3)	841.53 million (11)
Total	3.73 billion (100)	3.92 billion (100)	7.65 billion (100)
Financial costs	495.31 million (13.3)	2.04 billion (52)	2.54 billion (33.2)
**US**			
Direct medical cost	160.26 million (0.8)	4.45 billion (18.5)	4.60 billion (10.6)
Indirect medical cost	6.25 billion (32.5)	1.66 billion (7.0)	7.91 billion (18.3)
Well-being cost	5.86 billion (30.4)	6.72 billion (28.0)	12.58 billion (29.1)
Productivity cost	6.98 billion (36.3)	11.14 billion (46.5)	18.12 billion (42)
Total	19.25 billion (100)	23.98 billion (100)	43.23 billion (100)
Financial costs	13.39 billion (69.6)	17.25 billion (72)	28.99 billion (71)

^a^ To convert to US$, multiply by 1.14.

**Table 4.** Estimated Per-Annum Individual Cost According to Age-Related Macular Degeneration (AMD) Type and Cost Categories Across the Studied Countries

	Cost (% of total cost), €^a^			
	Geographic atrophy	Neovascular AMD		All AMD
**Bulgaria**				
Direct medical cost	306 (1.6)	3516 (17.5)		1911 (9.6)
Indirect medical cost	811 (4.2)	2095 (10.4)		1453 (7.3)
Well-being cost	16 910 (87)	12 923 (64.4)		14 916 (75.4)
Productivity cost	1461 (7.5)	1516 (7.6)		1489 (7.5)
Total	19 490 (100)	20 050 (100)		19 770 (100)
Financial costs	2579 (13.2)	7127 (35.5)		4853 (24.5)
**Germany**				
Direct medical cost	178 (0.9)	5028 (24.5)		2603 (13)
Indirect medical cost	751 (3.8)	2907 (14.1)		1829 (9)
Well-being cost	16 923 (86.7)	9866 (48.0)		13 395 (67)
Productivity cost	1658 (8.5)	2738 (13.3)		2198 (11)
Total	19 511 (100)	20 540 (100)		20 025 (100)
Financial costs	2587 (13.3)	10 673 (52)		6630 (33.1)
**US**				
Direct medical cost	327 (0.8)	9073 (18.5)		4700 (10.6)
Indirect medical cost	12 744 (32.5)	3385 (7.0)		8064 (18.3)
Well-being cost	11 948 (30.4)	13 702 (28.0)		12 825 (29.1)
Productivity cost	14 232 (36.3)	22 714 (46.5)		18 473 (42)
Total	39 251 (100)	48 874 (100)		44 063 (100)
Financial costs	27 303 (69.6)	35 172 (72)		31 237 (71)

^a^ To convert to US$, multiply by 1.14.

### Germany

The total economic burden of late-stage AMD was estimated to range between €1.4 billion ($1.6 billion) and €10.10 billion ($11.51 billion). Considering the midvalue prevalence rate, the total cost attributable was estimated at €7.6 billion ($8.6 billion). In both GA and nAMD, most of the cost (88% in GA and 48% in nAMD) was attributed to well-being. The financial costs incurred due to GA and nAMD were €495.31 million ($546.77 million) and €2.04 billion ($2.32 billion), respectively (Table 3). In GA, 98% of the total cost incurred due to the loss of productivity resulted from the impact on patients, while the remaining 2% of the cost was attributable to the impact on caregivers. In nAMD, 93% of the total cost attributed to the loss of productivity was due to the impact on patients, with caregivers contributing to the remaining 7% of the cost. Per-annum individual cost incurred due to GA and nAMD was estimated at €19 482 ($22 214) and €20 475 ($23 346), respectively (Table 4).

### US

The total economic burden of late-stage AMD was estimated to range between €8.3 billion ($9.5 billion) and €119.7 billion ($136.5 billion). Considering the midvalue prevalence rate, the total cost attributable was estimated at €43.2 billion ($49.4 billion). For both GA and nAMD, the greatest share of total costs was attributed to the loss of productivity. In nAMD, the loss of productivity accounted for 46% of the total cost, and in GA, it accounted for 36%. The financial costs incurred due to nAMD amounted to €17.2 billion ($19.7 billion) and totaled €13.4 billion ($15.3 billion) for GA (Table 3). In GA, 88% of the total cost incurred due to the loss of productivity resulted from the impact on patients, while the remaining 12% of the cost was attributable to the impact on caregivers. In nAMD, 99% of the total cost attributed to the loss of productivity was due to the impact on patients, with caregivers contributing to the remaining 1% of the cost. Per-annum individual costs incurred due to nAMD and GA were estimated at €48 895 ($55 752) and €39 250 ($44 755), respectively (Table 4).

### Cost Comparison Across Countries

The estimated costs of GA and nAMD showed considerable variation among the studied countries and across the 4 cost categories (Tables 3 and 4). Notable differences included higher direct medical costs for nAMD compared to GA across all countries. For indirect costs, well-being costs contributed the largest share in Bulgaria and Germany compared to productivity costs in the US. Moreover, indirect medical costs for GA were substantially higher in the US than in Bulgaria and Germany. Well-being costs were higher for GA than for nAMD across all countries. Productivity costs were much higher in the US than in Bulgaria and Germany for both GA and nAMD. In Bulgaria and Germany, financial costs accounted for 13% of the total costs for GA and 35% to 52% of the total costs for nAMD, respectively. However, in the US, financial costs represented a much higher proportion of the total costs for both GA (70%) and nAMD (72%).

## Discussion

This economic evaluation study provided an estimate of the economic burden of late-stage AMD across 3 countries—Bulgaria, Germany, and the US. The investigation incorporated numerous areas that could be affected by the disease, including the well-being of patients as well as the productivity of both patients and caregivers. The study demonstrated a relatively large economic burden of the late-stage AMD in those countries. Existing research has consistently highlighted the substantial financial burden associated with AMD, predominantly focusing on nAMD.^7,8,9,11,12,22,23,24^ A study by Cruess et al^22^ estimated the annual economic burden of nAMD in 2005 across Canada, France, Germany, Spain, and the United Kingdom to be between €671 million and €3.3 billion. Concurrently, the annual economic burden of AMD in the US for the same year was estimated to be nearly $30 billion.^25^ The mean annual cost per patient estimated in this study is higher than that indicated in prior reports.^22,26,27,28^ The inclusion of intangible costs, such as those related to reduced well-being, likely accounts for our higher estimates compared to previous research that largely concentrated on direct, indirect, and productivity-related expenses.^7,8,9,12,22,23,24,29,30^

We observed higher direct medical costs for nAMD compared with GA across all countries. This finding is reasonable given the fact that several treatment options were available for nAMD but not for GA at the time of conducting this research.

The main driver of costs associated with GA in Germany and Bulgaria was well-being. Well-being issues, such as depression and anxiety, were more prevalent among people with GA than in those with nAMD in these 2 countries. This was in contrast to patients in the US, where there was no meaningful difference in well-being cost between GA and nAMD. The reason for this finding is unclear but it could be related to differences in health care access, social support, or attitudes toward vision loss across the countries studied. Moreover, the difference in patient populations across countries may have affected the well-being results. Further research is needed to fully explain the discrepancy. Nevertheless, considering the costs incurred due to GA and nAMD together, the high well-being costs across all 3 countries suggests a substantial association of the disease on individuals’ reported vision-related quality of life.

In the US, the greatest share of costs for both GA and nAMD was attributed to the loss of productivity. This may signify a greater impact of the disease in the working-age population in the US compared to the other countries studied. The per-annum, per-individual cost due to loss of productivity estimated in this study is in line with previous estimates reported in the literature, which ranged from $1395 to $55 180.^31^ In Bulgaria and Germany, for both GA and nAMD, most of the productivity cost (93%-99%) was contributed by individuals with AMD, with a fraction of the cost contributed by the caregivers. However, in the US, particularly for GA, a sizeable proportion of productivity cost (12%) was attributed to caregivers’ loss of productivity. This could mean that a considerable proportion of people living with GA in the US are severely impacted by the disease, leading to loss of independence and hence requiring greater caregiver support, which confirms the findings in a previous study in the US.^11^ This finding suggests that there is an immediate need to enhance caregiver support and promote workplace accommodations for patients with AMD across countries.

One potentially novel aspect of this study was to quantify well-being associated with the disease in terms of monetary value. While this approach is not widely used in literature, we believe that incorporating these costs is essential to understand the full burden of the disease. We demonstrated that the cost attributed to well-being accounted for a considerable proportion of the total burden. This implies that in addition to managing their physical condition, the psychological well-being and the emotional needs of people living with AMD and caregivers also need to be addressed. This finding aligns with a prior study in the UK^32^ that highlights the need of such services in the sight care pathway for patients and caregivers.

This study found that the estimated annual economic burden associated with late-stage AMD was commensurate with the economic burdens imposed by other non–life-threatening conditions, such as Parkinson disease and obesity.^33,34^ However, there has been a substantial disparity in funding allocation to AMD research from governmental and state funding agencies.^34,35^ Moreover, there is a lack of a broader focus on eye and vision research within prioritized research areas, particularly in Europe.^36^

### Limitations

Our study has some limitations that warrant acknowledgment. First, the patient survey data were based on a relatively small sample of participants in each country; therefore, the model may have over- or underestimated certain cost parameters. More survey data would have improved the accuracy and generalizability of the study results. However, key variables in our economic model, such as prevalence of anxiety and depression^37^ and the magnitude of productivity loss in people living with AMD,^31^ aligned with literature-reported figures. Therefore, we believe that the present study provides a valid estimate. Second, we estimated the association of the disease with well-being in monetary terms based on the principles of disability-adjusted life years and quality-adjusted life years. In the absence of disease-specific quality-adjusted life year values for AMD in some countries, we had to make assumptions that could have over- or underestimated the well-being cost category. Despite these constraints, our study provides a picture of the substantial direct, indirect, and intangible costs imposed by late-stage AMD.

## Conclusions

In conclusion, late-stage AMD imposes a substantial economic burden akin to other common non–life-threatening diseases, such as Parkinson disease and obesity. To our knowledge, this is the first study to comprehensively demonstrate the magnitude of the costs beyond the direct and indirect medical expenditures to also encompass well-being and productivity costs associated with late-stage AMD. These findings suggest an urgent need to identify strategies to minimize the costs and impact for patients, caregivers, and society. Raising awareness and improving early detection and access to vision rehabilitation services could help mitigate some of the indirect costs imposed by vision loss due to late-stage AMD. Access to current and future therapies for late-stage AMD, including both neovascular AMD and GA, holds promise for reducing the projected increase in AMD burden amid global aging populations.

## Notes

Supplement 1. eTable. 1 Details of the input data obtained from the literature to estimate direct medical costs eTable. 2 Details of the input data obtained from the survey to estimate direct medical costs eTable. 3. Details of the input data obtained from the literature to estimate indirect medical costs eTable. 4. Details of the input data obtained from the survey to estimated indirect medical costs eTable. 5. Details of the input data obtained from the literature to estimate wellbeing costs eTable. 6. Details of the input data obtained from the survey to estimate wellbeing costs eTable. 7. Details of the input data obtained from the literature to estimate productivity costs. eTable. 8. Details of the input data obtained from the survey to estimate productivity costs. eFigure 1. Schematic overview of economic burden calculation eFigure 2. Schematic overview of direct medical cost calculation. *Direct costs for those living with GA only account for imaging eye exams eFigure 3. Schematic overview of indirect medical costs calculation. # - Number eFigure 4. Schematic overview of well-being costs calculation. DALY – Disability Adjusted Life Years, QALY – Quality Adjusted Life Years eFigure 5. Schematic overview of productivity loss costs eReferences eAppendix 1. Patient Survey eAppendix 2. Caregivers Survey

Supplement 2. Data sharing statement
