# Supplementary material for: Economic Burden of Late-Stage Age-Related Macular Degeneration in Bulgaria, Germany, and the US
Source: JAMA Ophthalmol. 2024 Oct 31;142(12):1123–30. doi: 10.1001/jamaophthalmol.2024.4401 (PMC12316190; doi:10.1001/jamaophthalmol.2024.4401)
Supplement: Supplement 1. — eTable. 1 Details of the input data obtained from the literature to estimate direct medical costs eTable. 2 Details of the input data obtained from the survey to estimate direct medical costs eTable. 3. Details of the input data obtained from the literature to estimate indirect medical costs eTable. 4. Details of the input data obtained from the survey to estimated indirect medical costs eTable. 5. Details of the input data obtained from the literature to estimate wellbeing costs eTable. 6. Details of the input data obtained from the survey to estimate wellbeing costs eTable. 7. Details of the input data obtained from the literature to estimate productivity costs. eTable. 8. Details of the input data obtained from the survey to estimate productivity costs. eFigure 1. Schematic overview of economic burden calculation eFigure 2. Schematic overview of direct medical cost calculation. *Direct costs for those living with GA only account for imaging eye exams eFigure 3. Schematic overview of indirect medical costs calculation. # - Number eFigure 4. Schematic overview of well-being costs calculation. DALY – Disability Adjusted Life Years, QALY – Quality Adjusted Life Years eFigure 5. Schematic overview of productivity loss costs eReferences eAppendix 1. Patient Survey eAppendix 2. Caregivers Survey [file jamaophthalmol-e244401-s001.pdf]

Supplemental Online Content

Paudel N, Brady L, Stratieva P, et al. Economic Burden of Late-Stage Age-Related Macular Degeneration in Bulgaria, Germany, and the US. *JAMA Ophthalmol*. Published online October 31, 2024. doi:10.1001/jamaophthalmol.2024.4401

eTable. 1 Details of the input data obtained from the literature to estimate direct medical costs

eTable. 2 Details of the input data obtained from the survey to estimate direct medical costs

eTable. 3. Details of the input data obtained from the literature to estimate indirect medical costs

eTable. 4. Details of the input data obtained from the survey to estimated indirect medical costs

eTable. 5. Details of the input data obtained from the literature to estimate wellbeing costs

eTable. 6. Details of the input data obtained from the survey to estimate wellbeing costs

eTable. 7. Details of the input data obtained from the literature to estimate productivity costs.

eTable. 8. Details of the input data obtained from the survey to estimate productivity costs.

eFigure 1. Schematic overview of economic burden calculation

eFigure 2. Schematic overview of direct medical cost calculation. *Direct costs for those living with GA only account for imaging eye exams

eFigure 3. Schematic overview of indirect medical costs calculation. # - Number

eFigure 4. Schematic overview of well-being costs calculation. DALY – Disability Adjusted Life Years, QALY – Quality Adjusted Life Years

eFigure 5. Schematic overview of productivity loss costs

eReferences

eAppendix 1. Patient Survey

© 2024 Paudel N et al. *JAMA Ophthalmology.*

eAppendix 2. Caregivers Survey

This supplemental material has been provided by the authors to give readers additional information about their work.

© 2024 Paudel N et al. *JAMA Ophthalmology.*

Table 1 Details of the input data obtained from the literature to estimate direct medical costs

Variables	Bulgaria	Germany	USA
Visual Field testing	€76.65[1]	€17.35[2]	€73.50[3]
Eye exam with photos (fundus or optic nerve photography)	€12.77[1]	€13.55 [2]	€98.18[3]
Ocular coherence tomography (OCT)	€338.57[1]	€44.95 [2]	€51.25[3]
Fluorescein angiography (FA)	€25.54[1]	€48.85 [2]	€185.54[3]
Number of Mean visits p/a - Visual Field testing	8[1]	4[4]	4[4]
Number Mean visits p/a - Eye exam with photos (fundus or optic nerve photography)	1[1]	2[4]	2[4]
Number Mean visits p/a - Ocular coherence tomography (OCT)	7[1]	2[2]	2[4]
Number Mean visits p/a - Fluorescein angiography (FA)	1[1]	2 [4]	2[4]
Lucentis	-	€69.40[5]	€293.99[5]
Beovu	€600.97[1]	€27.10[5]	€196.36[5]
Eylea	€779.93[1]	€89.90[5]	€102.51[5]
Avastin	€1,009.22[1]	€97.70[5]	€371.08[5]
Share use of Lucentis	0[6]	0.17[6]	0.09[6]
Share use of Beovu	0.2[6]	0.13[6]	0.01[6]
Share use of Eylea	0.2[6]	0.2[6]	0.3[6]
Share use of Avastin	0.6[6]	0.5[6]	0.6[6]

© 2024 Paudel N et al. *JAMA Ophthalmology.*

Table 2 Details of the input data obtained from the survey to estimate direct medical costs

Variables	Bulgaria	Germany	USA
Number of Eye care visits (nAMD)	8.4	8.8	8.5
Number of Eye care visits (GA)	2.7	5.7	3.2
Number of Treatments/ surgeries per year	4.7	6.8	9.5

Table 3. Details of the input data obtained from the literature to estimate indirect medical costs

Variables	Bulgaria	Germany	USA
Emergency day visit cost	€45.97[1]	€21.69[7]	€605.13[8]
Inpatient stay cost	€463.01[1]	€1,612[9]	€1,374.04[8]
Home help	€4,103.39[1]	€13,726[9]	€48,157.82[10]
Nursing home care	€8,393.30[1]	€14,248.80[9]	€87,288.61[10]

© 2024 Paudel N et al. *JAMA Ophthalmology.*

Table 4. Details of the input data obtained from the survey to estimated indirect medical costs

Variables	Bulgaria	Germany	USA
% Cared for by children	68%	32%	69%
% Cared for by spouse/ domestic partner	32%	66%	29%
# of accidents per person p/a (nAMD)	1.5	0.3	1.3
# of accidents per person p/a (GA)	2.2	0.4	1.3
% of accidents not requiring treatment (nAMD)	33%	80%	91%
% of accidents not requiring treatment (GA)	63%	80%	83%
% of accidents requiring treatment (ED) (nAMD)	67%	6%	9%
% of accidents requiring treatment (ED) (GA)	25%	6%	0%
% of accidents requiring treatment (hospital stay) (nAMD)	0%	14%	0%
% of accidents requiring treatment (hospital stay) (GA)	13%	14%	17%
Travel time to appointments (nAMD)	2	1.58	0.55
Travel time to appointments (GA)	1.94	2.1	1.13
Number of appointments per year (nAMD)	8.4	8.8	8.5
Number of appointments per year (GA)	2.7	5.7	3.2
% requiring home modifications (nAMD)	40%	16%	83%
% requiring home modifications (GA)	94%	50%	63%
Share in home care (nAMD)	10%	3%	0%
Share in home care (GA)	0%	0%	7%
Share gaining formal care (nAMD)	0%	0%	3%
Share gaining formal care (GA)	0%	0%	0%

© 2024 Paudel N et al. *JAMA Ophthalmology.*

Table 5. Details of the input data obtained from the literature to estimate wellbeing costs

Variables	Bulgaria	Germany	USA
Willingness-to-pay QALY	€49,971.46[11]	€58,533[12]	€43,850[13]
WHO QALY – Anxiety[14]	0.091	0.091	0.091
WHO QALY – Depression[14]	0.302	0.302	0.302
WHO QALY – Low vision[14]	0.245	0.245	0.245

Table 6. Details of the input data obtained from the survey to estimate wellbeing costs

Variables	Bulgaria	Germany	USA
% Reporting anxiety (nAMD)	50%	16%	53%
% Reporting anxiety (GA)	71%	30%	50%
% reporting blindness (nAMD)	0%	0%	0%
% reporting blindness (GA)	0%	0%	0%
% Reporting depression (nAMD)	30%	19%	47%
% Reporting depression (GA)	67%	46%	48%
% reporting low vision (nAMD)	50%	39%	50%
% reporting low vision (GA)	76%	70%	47%

© 2024 Paudel N et al. *JAMA Ophthalmology.*

Table 7. Details of the input data obtained from the literature to estimate productivity costs.

Variables	Bulgaria	Germany	USA
Average travel costs (per hour)	€4.19[15]	€12.40[15]	€14.30[16]
Average annual gross salary [17]	€8,377	€37,052	€58,325
Productivity per hour[18]	€5.22	€27.82	€33
Average annual working hours[19]	1,605	1,332	1,767
Value of leisure time	€4.19[15]	€12.40[15]	€14.30[16]
Share of work hours lost due to VI[20]	0.20	0.20	0.20

Table 8. Details of the input data obtained from the survey to estimate productivity costs.

**Variables**	**Bulgaria**	**Germany**	**USA**
% of people in employment (GA)	100%	83%	91%
% of people in employment (nAMD)	100%	100%	100%
% Job loss as result of GA	13%	0%	36%
% Job loss as result of nAMD	20%	3%	60%
% experience job reduction as result of GA	88%	75%	14%
% experience job reduction as result of nAMD	90%	73%	40%
Carer - % job loss (nAMD)	0%	0%	0%
Carer - % job loss (GA)	0%	0%	0%
Carer - % job reduction due to care (nAMD)	0%	13%	17%
Carer - % job reduction due to care (GA)	25%	0%	25%
Carer - % hours reduced due to care (nAMD)	0%	5%	3%

© 2024 Paudel N et al. *JAMA Ophthalmology.*

Carer - % hours reduced due to care (GA)	3%	0%	18%
% Providing transport to appointment (nAMD)	43%	87%	67%
% Providing transport to appointment (GA)	75%	80%	100%
% Providing transport during work hours (nAMD)	29%	27%	64%
% Providing transport during work hours (GA)	44%	20%	50%
% Providing transport during non-work hours (nAMD)	14%	60%	2%
% Providing transport during non-work hours (GA)	31%	60%	50%
Number of journeys per annum (nAMD)	4	11	11
Number of journeys per annum (GA)	2	4	4

© 2024 Paudel N et al. *JAMA Ophthalmology.*

Figure 1. **Schematic overview of economic burden calculation**

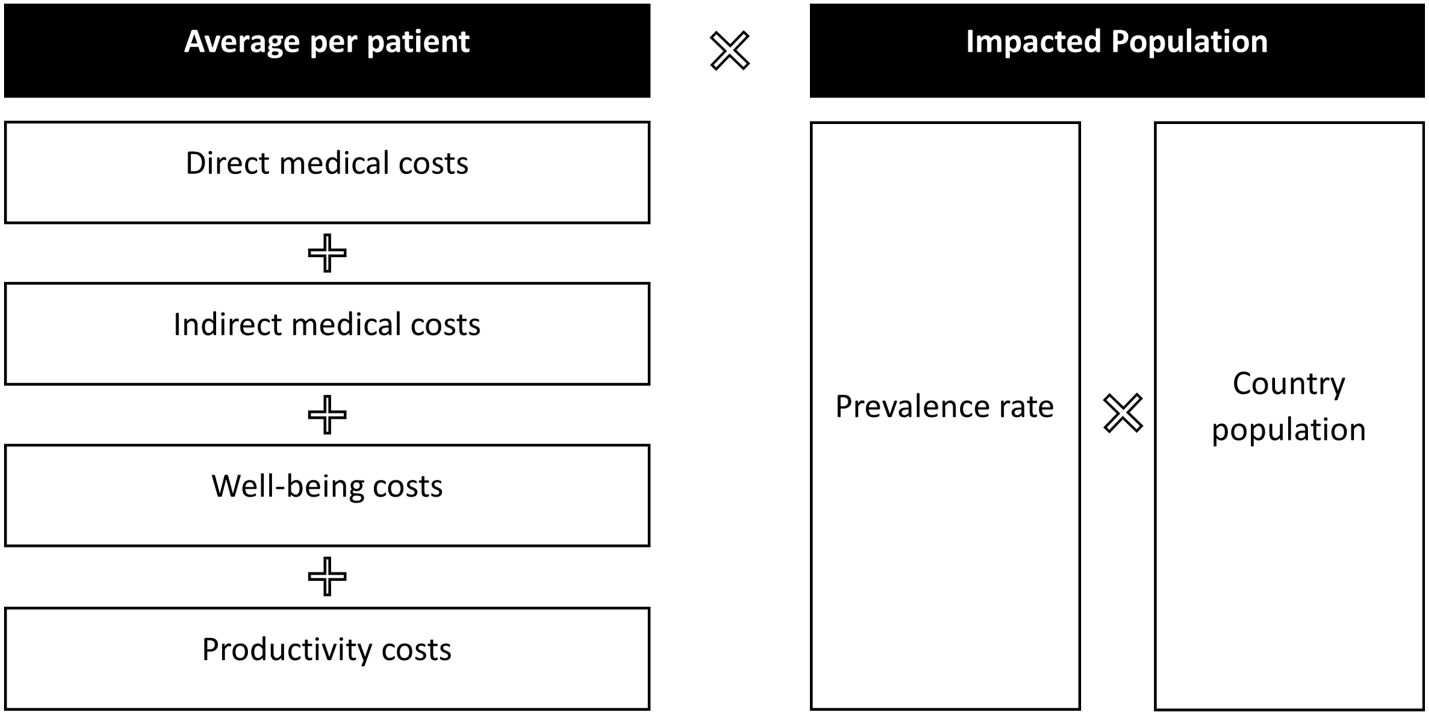

© 2024 Paudel N et al. *JAMA Ophthalmology.*

Figure 2. Schematic overview of direct medical cost calculation. *Direct costs for those living with GA only account for imaging eye exams

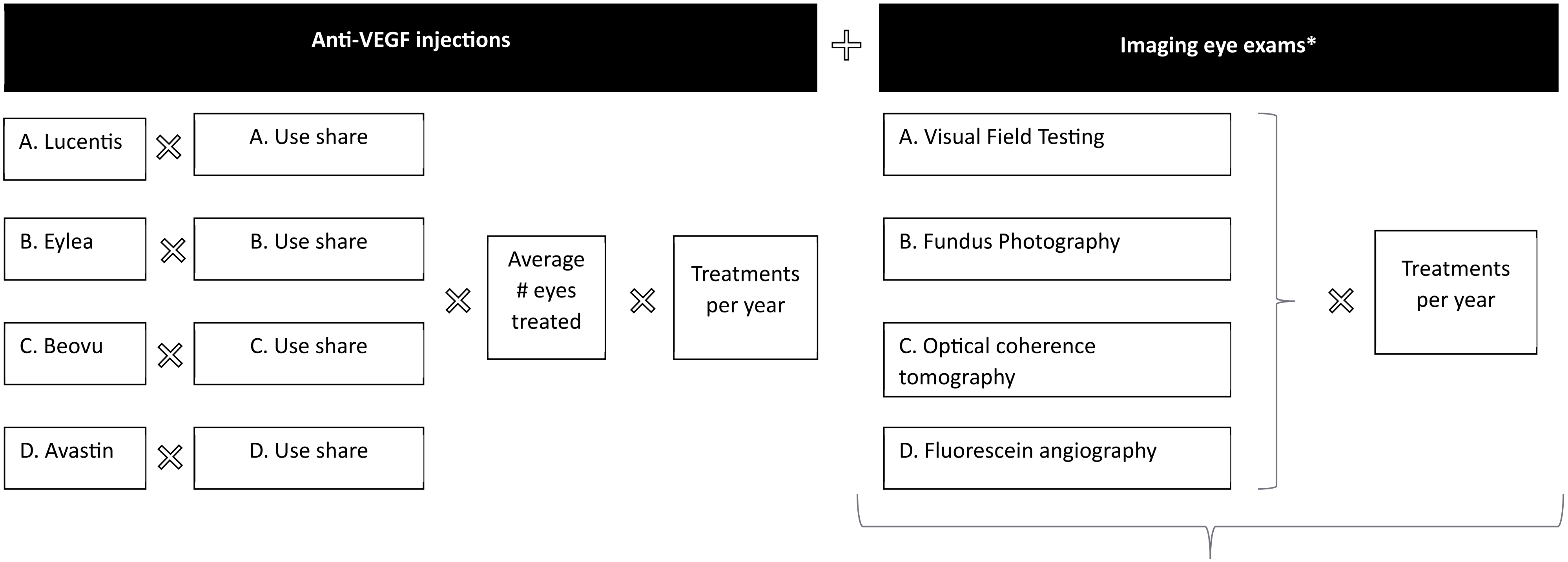

© 2024 Paudel N et al. *JAMA Ophthalmology.*

Figure 3. Schematic overview of indirect medical costs calculation. #- Number

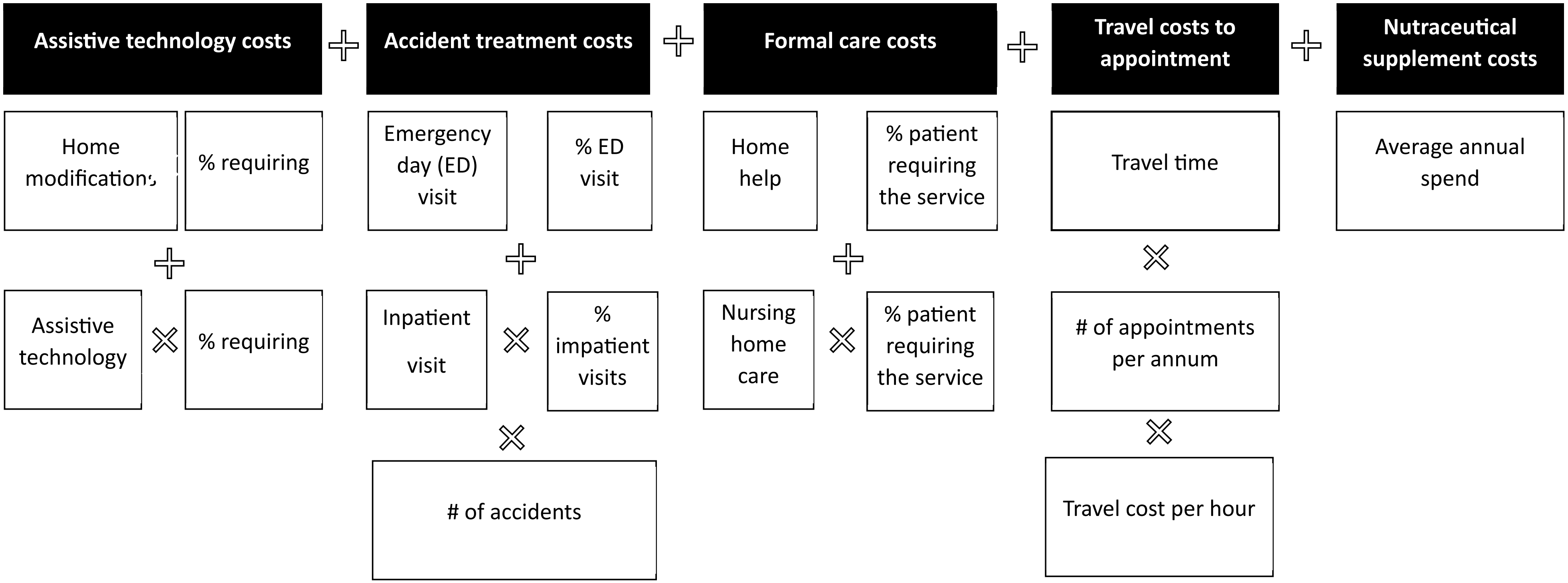

© 2024 Paudel N et al. *JAMA Ophthalmology.*

Figure 4. Schematic overview of well-being costs calculation. DALY – Disability Adjusted Life Years, QALY – Quality Adjusted Life Years

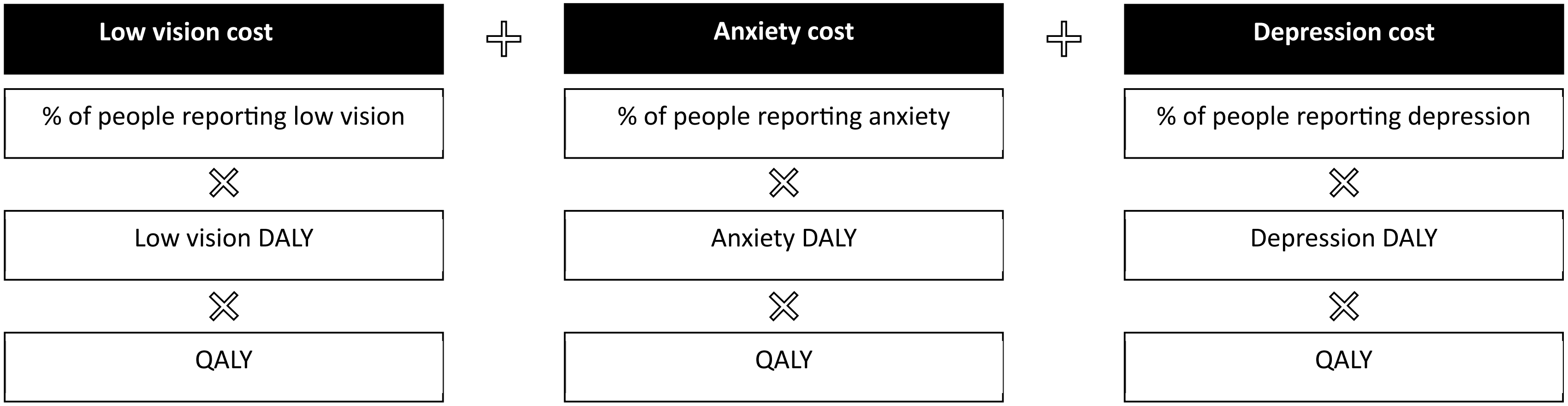

© 2024 Paudel N et al. *JAMA Ophthalmology.*

Figure 5. Schematic overview of productivity loss costs

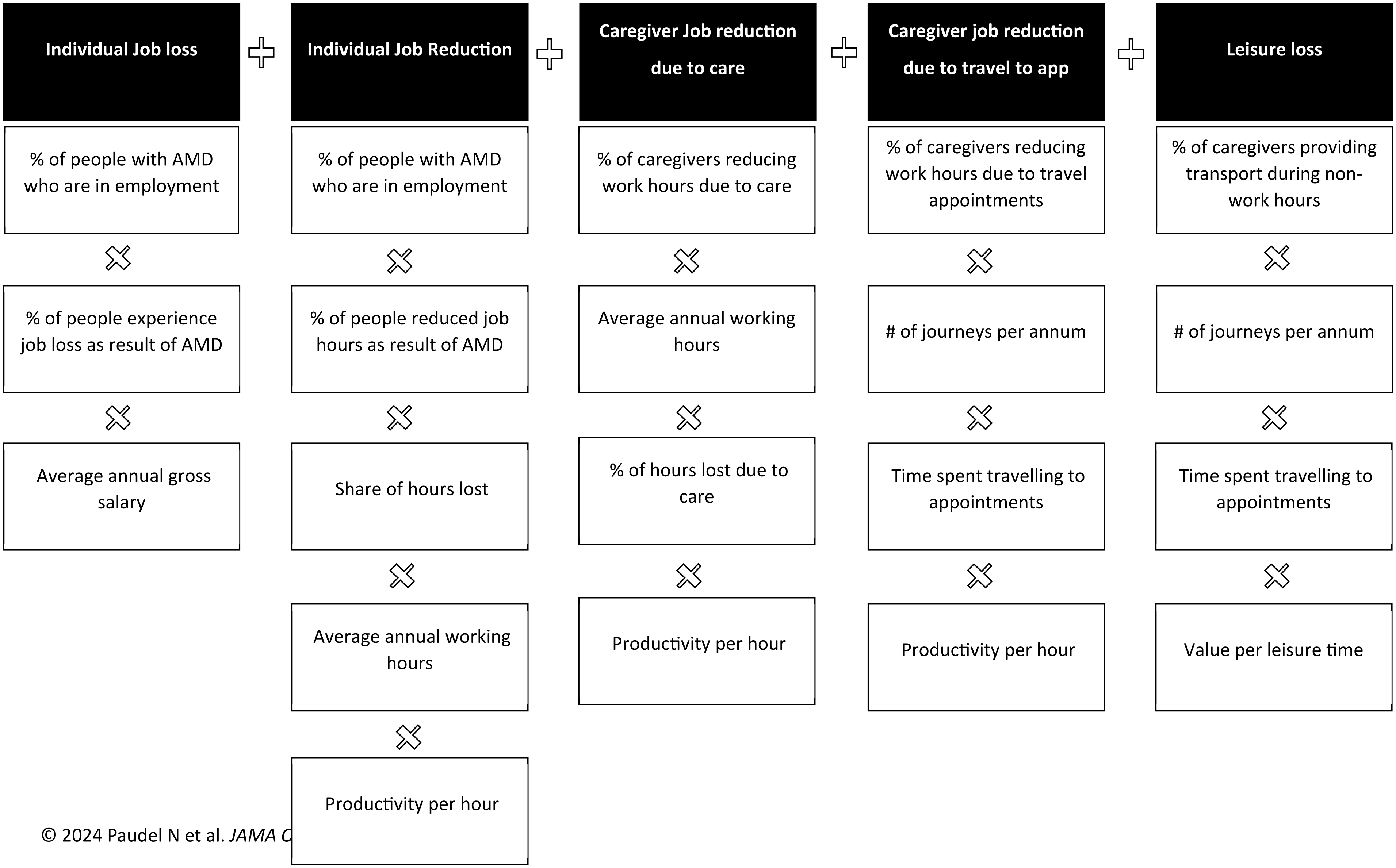

© 2024 Paudel N et al. *JAMA C*[illegible]

References:

1. National Health Insurance Fund. Accessed March 1, 2024. https://www.nhif.bg/en

2. KBV - Kassenärztliche Bundesvereinigung. Accessed March 1, 2024. https://www.kbv.de/html/index.php

3. CMS-1736-FC | CMS. Accessed February 16, 2024. https://www.cms.gov/medicaremedicare-fee-service-paymenthospitaloutpatientppshospital-outpatient-regulations-and-notices/cms-1736-fc

4. Fundus Photography - Medical Clinical Policy Bulletins | Aetna. Accessed March 1, 2024. https://www.aetna.com/cpb/medical/data/500_599/0539.html

5. Global Data. Accessed February 16, 2024. https://www.globaldata.com/

6. Consultation with relevant practitioners in respective countries.

7. KBV - EBM Suche. Accessed February 16, 2024. https://www.kbv.de/html/13259.php

8. Howland J, Shankar KN, Peterson EW, Taylor AA. Savings in acute care costs if all older adults treated for fall-related injuries completed matter of balance. *Inj Epidemiol*. 2015;2(1). doi:10.1186/S40621-015-0058-Z

9. Federal Ministry of Health. Accessed April 2, 2024. https://www.bundesgesundheitsministerium.de/en/

10. Cost of Long Term Care by State | Cost of Care Report | Genworth. Accessed April 2, 2024. https://www.genworth.com/aging-and-you/finances/cost-of-care

11. Iskrov G, Greenberg D, Yakimov I, Cholakova H, Stefanov R. What Is the Value of Innovative Pharmaceutical Therapies in Oncology and Hematology? A Willingness-to-Pay Study in Bulgaria. *Value Health Reg Issues*. 2019;19:157-162. doi:10.1016/J.VHRI.2019.03.005

12. Himmler S, Stöckel J, van Exel J, Brouwer WBF. The value of health—Empirical issues when estimating the monetary value of a quality-adjusted life year based on well-being data. *Health Econ*. 2021;30(8):1849-1870. doi:10.1002/HEC.4279

13. Institute for Clinical and Economic Review. 2020-2023 value assessment framework. Accessed April 2, 2024. icer.org/wp-content/uploads/2020/10/ICER_2020_2023_VAF_102220.pdf

14. Abbafati C, Abbas KM, Abbasi M, et al. Global burden of 369 diseases and injuries in 204 countries and territories, 1990–2019: a systematic analysis for the Global Burden of Disease Study 2019. *The Lancet*. 2020;396(10258):1204-1222. doi:10.1016/S0140-6736(20)30925-9

© 2024 Paudel N et al. *JAMA Ophthalmology.*

15. Wardman M, Chintakayala VPK, de Jong G. Values of travel time in Europe: Review and meta-analysis. *Transp Res Part A Policy Pract*. 2016;94:93-111. doi:10.1016/J.TRA.2016.08.019

16. US Department of Transportation. Revised Departmental Guidance on Valuation of Travel Time in Economic Analysis | US Department of Transportation. Accessed February 16, 2024. https://www.transportation.gov/office-policy/transportation-policy/revised-departmental-guidance-valuation-travel-time-economic

17. Oxford Economics. Accessed February 16, 2024. https://www.oxfordeconomics.com/

18. Ernst and Young. *Ernst & Young Analysis*. 2022.

19. Employment - Hours worked - OECD Data. Accessed April 7, 2024. https://data.oecd.org/emp/hours-worked.htm

20. Retina International. *Economic Burden of Late-Stage Age Related Macular Degeneration in Bulgaria, Germany and the USA.* 2022.

© 2024 Paudel N et al. *JAMA Ophthalmology.*

Patient Survey

Throughout the survey the word condition is utilized to refer to either Geographic Atrophy or Wet-Age-related Macular Degeneration as selected in question 1.

Module 1 Demographic Information

1. Which of the following best describes how you are responding to this survey?
- I consent to participate in this survey as a person living with Geographic Atrophy
- I consent to participate in this survey as a person living with wet Age-related Macular Degeneration
- I do not consent to participate in this Survey-Survey cannot be completed

2. Are you:
- Male
- Female
- Other

3. What is your age range?
- 55-59
- 60-64
- 65-69
- 70-74
- 75-79
- 80-84
- 85-90
- 90-95
- 95+

4. What country do you live in?

© 2024 Paudel N et al. *JAMA Ophthalmology.*

5. Would you describe the area you live in as?

- A City
- A Town
- A Village
- A Rural Area/ The Countryside

Module 2 Your Condition

6. Which of these best describes the vision (if any) you have remaining?
   - No vision
   - Light perception only (or shadows only)
   - Some useful central vision
   - Good central vision
   - Some useful peripheral vision
   - Good peripheral vision
   - I still have good overall vision
   - Other – please specify

7. Are you registered as legally blind, or partially sighted with your government or health service/health system?
   - Yes

© 2024 Paudel N et al. *JAMA Ophthalmology.*

- No
- Unsure
- Prefer not to say

8. How long ago were you diagnosed with your condition?
   - More than 6 months ago
   - More than 1 year ago
   - More than 2 years ago
   - More than 3 years ago
   - More than 5 years ago
   - More than 10 years ago
   - More than 15 years ago
   - More than 20 years ago

9. Did you notice a change in vision prior to your diagnosis?
   - Yes
   - No
   - I don't know

10. If yes is selected.
    Approximately how much time passed between realizing there was a change in your vision and acquiring a diagnosis?
    - Free Text

11. Is there family history of your condition?
    - Yes
    - No
    - Unsure
    - Prefer not to say

© 2024 Paudel N et al. *JAMA Ophthalmology.*

Module 3 Impact on living

12. Has your condition impacted on your work in any way?
- Yes
- No
- Prefer Not to Say
- Already Retired
- Already not working

13. If yes is selected the following question is presented.
    Please select all that are relevant to you. Has your condition?
- Forced you to retire early
- Forced you to reduce hours
- Forced you to change jobs
- Increased your absence from work

14. Has your condition impacted on your ability to drive?
- Yes- driving is increasingly more difficult
- Yes- I can no longer drive
- No
- Prefer not to say
- Not applicable

15. Has your condition led you to relocate your primary residence?
- Yes- temporarily
- Yes-permanently
- No
- Prefer not to say

© 2024 Paudel N et al. *JAMA Ophthalmology.*

16. If yes is selected the following question is presented.

Have you moved residence to?

- A family members house/home
- Residential care/nursing home
- Other (please specify)

17. Have you had to invest in home modifications at your primary residence due to your condition?
- Yes
- No
- Prefer not to say

18. If yes is selected the following question is presented.

Please select all modifications you have made at your primary residence.

- High vision lamps
- Bathroom modifications
- Kitchen modifications
- Customized clocks/ timers
- Ergonomic adaptations
- Use of lighting during the daytime
- Other (please specify)

19. Approximately how much have you spent in the **past year** on modifications you have made to your primary residence?
    - Free Text

20. In the **past 4 weeks** have you utilized any assistive resources or visual aids to support living with your condition? Please select all that apply.
    - White cane
    - Guide dog

© 2024 Paudel N et al. *JAMA Ophthalmology.*

- Digital speech to text converter
- Screen reader
- Magnifier
- Phone, tablet, or laptop with applications or modifications to support your low vision needs
- Audiobooks
- Other-please specify
- None

21. Approximately how much have you spent in the **past 4 weeks** on any assistive resources to support living with your condition?
    - Free Text

22. How frequently do you visit an eye care professional such as an optometrist, ophthalmologist or eye doctor to monitor or treat your condition? Please select the most relevant answer.
    - Once per month
    - Once every 2 months
    - Once every 3 months
    - Once every 4 months
    - Once every 6 months
    - Once per year
    - More than once per year

23. How far do you have to travel for appointments to monitor or treat your condition?
    - Less than 10 miles (or kilometers depending on region)
    - Between 10 and 20 miles
    - Between 20 and 50 miles
    - More than 50 miles

24. How do you travel most often to your appointments to monitor or treat your condition? Please select the most appropriate answer.
    - Walking
    - Public Transport

© 2024 Paudel N et al. *JAMA Ophthalmology.*

- My Own Private Car
- Someone else’s private car
- Taxi (or equivalent)

25. Does someone accompany you to your appointments to monitor or treat your condition?
- No I travel and attend my appointments on my own
- Yes, for all of my appointments
- Yes, for about three quarters of my appointments
- Yes, for about half of my appointments
- Yes, for about one quarter of my appointments

26. Approximately how much **per year** do you spend on appointments with eye care professionals?
- Free Text
- Prefer not to say

27. Approximately how much **per year** do you spend on medications or supplements for your condition?
- Free Text
- Prefer not to say

28. How much do you spend **per year** on low vision rehabilitation services?
- Free Text
- Prefer not to say

29. Has your condition led you to have an accident in the **past year** such as a slip, trip, fall, burn, car/ bicycle/vehicle crash?
    - Yes
    - No
    - Prefer not to say

30. If yes is selected.
    How many accidents in the **past year**? (Free Text Box)

© 2024 Paudel N et al. *JAMA Ophthalmology.*

31. What was the impact?
    - No damage or injury
    - Minor injury- did not require hospital treatment
    - Major injury-requiring emergency day care
    - Major injury-requiring hospital stay

32. Does your condition require you to receive additional assistance for any of the following daily tasks? Please select all that apply.
    - Housekeeping- cleaning the house/doing the laundry
    - Gardening
    - Personal hygiene and/or grooming
    - Clothes shopping
    - Grocery shopping
    - Shopping for medical supplies or medications
    - Transport to or from medical appointments
    - Reading the mail or any personal administration documents to you
    - Other-please specify
    - No I do not receive any assistance for any of the above tasks

33. If no is selected this question is skipped.
    Who provides this assistance to you? Please select all that apply.
    - My spouse or partner
    - My child/children/grandchildren
    - My brother/sister
    - My niece/nephew
    - My neighbor or friend
    - A formal care provider who is paid
    - Nursing care provider

© 2024 Paudel N et al. *JAMA Ophthalmology.*

- Other-please specify

34. Does your condition prevent or restrict you from enjoying any hobbies or interests you had prior to developing your condition? Please select all that apply.
    - Yes- watching sport
    - Yes- playing sport
    - Yes-watching movies
    - Yes-playing card games
    - Yes-playing board games
    - Yes- travelling
    - Yes- cooking
    - Yes-socializing
    - Yes- driving
    - Yes- cultural activities
    - Yes- religious activities
    - Yes- Other- please specify
    - No

35. Has your condition caused you to experience any of the following?

- Anxiety
- Depression
- Social isolation
- Financial stress
- Loss of confidence
- Loss of independence
- None of the above
- Prefer not to say

© 2024 Paudel N et al. *JAMA Ophthalmology.*

36. Have you sought professional support or guidance to help with any of the experiences in Question 35, for example anxiety, depression, social isolation, stress? Professional support may come from a counsellor, a psychologist, psychiatrist, or therapist.
    - Yes
    - No
    - I am waiting for an appointment
    - Prefer not to say
    - Other- free text box

37. If yes is selected:
    Approximately how much do you spend **per year** on professional support or guidance to help you manage any of the experiences referred to in Question 35 for example anxiety, depression, social isolation, stress?
    - Free Text

38. Do you live with any of the following other conditions? Please select all that apply.
    - Diabetes
    - Renal disease (Kidney disease)
    - Dementia (this includes Alzheimer's Disease)
    - Congestive Heart Failure
    - Stroke
    - Pulmonary conditions
    - Parkinson's Disease
    - Multiple Sclerosis
    - Osteoporosis
    - Cancer
    - Deafness
    - Other- please specify

39. Do you live with any of the following other vision complications?
    - Refractive Error myopia (near-sightedness), hyperopia (farsightedness), astigmatism (distorted vision at all distances), and presbyopia (loss of the ability to focus up close)

© 2024 Paudel N et al. *JAMA Ophthalmology.*

- Diabetes Related Retinopathy
- Diabetes Related Macular Edema
- Glaucoma
- Cataract
- Uveitis
- Other- please specify

Caregivers Survey:

Throughout the survey the word condition is utilized to refer to either Geographic Atrophy or Wet-Age-related Macular Degeneration as selected in question 1.

1. Which of the following best describes how you are responding to this survey?

- I consent to participate in this survey as a person who provides care and/or support and/or assistance to a person living with Geographic Atrophy (referred to as the care recipient throughout this survey)
- I consent to participate in this survey as a person who provides care and/or support and/or assistance to a person living with wet Age-related Macular Degeneration (referred to as the care recipient throughout this survey)
- I do not consent to participate in this Survey-Survey cannot be completed

2. Are you:

- Male
- Female
- Other

3. What is your age range?

- 18-24
- 25-34

© 2024 Paudel N et al. *JAMA Ophthalmology.*

- 35-44
- 45-54
- 55-64
- 65-74
- 75-84
- 85-94
- 95+

4. What country do you live in?

5. Would you describe the area you live in as?

- A City
- A Town
- A Village
- A Rural Area/ The Countryside

6. Is the person you provide care to?
    - Your spouse
    - Your parent
    - Your brother or sister
    - Your aunt or uncle
    - Your neighbour or friend
    - Other- please specify

7. How long have you been providing care to the care recipient? Please select the most appropriate answer.
    - More than 6 months
    - More than 1 year

© 2024 Paudel N et al. *JAMA Ophthalmology.*

- More than 2 years
- More than 3 years
- More than 5 years
- More than 10 years
- More than 15 years
- More than 20 years

8. What types of care and/or support and/or assistance do you provide to the care recipient with respect to their daily tasks and activities as a result of their vision? Please select all that apply.
    - Housekeeping- cleaning the house/doing the laundry
    - Gardening
    - Personal hygiene and/or grooming
    - Clothes shopping
    - Grocery shopping
    - Shopping for medical supplies or medications
    - Transport to or from medical appointments
    - Transport to or from social events
    - Transport to or from cultural events
    - Transport to or from religious services or events
    - Reading the mail or any personal administration documents to the care recipient
    - Providing care in the form of managing the health of care recipient
    - Providing care in the form of sourcing and providing information to the care recipient about their health and healthcare
    - Other- please specify

9. How much of your own money did you spend in the **past 4 weeks** providing care in the form of **driving** to complete the daily tasks described in Question 8?
    - Free Text

10. How much of your own money did you spend in the **past 4 weeks** providing care in the form of the daily tasks described in Question 8? **Please exclude any driving related costs.**

© 2024 Paudel N et al. *JAMA Ophthalmology.*

- Free Text

11. Did you take any time off work to provide care in the form of the daily tasks described in Question 8 in the **past 4 weeks**?
    - Yes- please specify how much time off work
    - No

12. Do you provide transport to the care recipient for their eye healthcare appointments?
    - Yes
    - No

13. If yes selected.
    How often do you provide transport each month or year for your care recipients eye healthcare appointments?
    Free Text for each month
    Free Text for each year

14. Approximately how far do you travel in total to support the care recipient for eye healthcare appointments? Please include the distance travelled from your location to that of the care recipient. Please also include the return journey.
    - Less than 10 miles/ kilometers
    - Between 10 and 20 miles/ kilometers
    - Between 20 and 50 miles/ kilometers
    - More than 50 miles/ kilometers

15. Do you take time off work to attend appointments with the care recipient specifically for monitoring or treating their eye health?
    - Yes, for all appointments
    - Yes, for about three quarters of appointments
    - Yes, for about half of appointments
    - Yes, for about one quarter of appointments
    - No, I do not take time off work to attend appointments

© 2024 Paudel N et al. *JAMA Ophthalmology.*

16. Has providing care led to any of the following?
    - Reduced working hours- if selected- how many hours less **per month** do you work?
    - Changing job
    - Increased absence from work- if selected-how many hours less **per month** do you work
    - Quitting work
    - None of the above
    - Prefer not to say

17. Have you had to provide financial assistance to the care recipient in the form of a gift that you do not expect returned?
    - Yes
    - No

18. If yes is selected.
    How much of your own money do you spend supporting the care recipient each month?
    - Free Text

19. Does your government or health service/health system provide any support to you as a care provider to someone living with a vision impairment?
    - Yes- Please specify
    - No
    - Prefer not to say

20. Has providing care led to any of the following?
    - Anxiety
    - Depression
    - Social isolation
    - Reduced time with your family
    - Reduced time with your friends
    - Reduced time for your leisure, hobbies and interests

© 2024 Paudel N et al. *JAMA Ophthalmology.*

- Reduced time for your health care
- Feelings of guilt
- Financial stress
- Loss of confidence
- Caused some strain on the relationship between you and the care recipient
- Caused some improvement on your relationship with the care recipient
- Other- please specify
- None of the above
- Prefer not to say

21. Have you sought professional support or guidance to help with any of the experiences in Question 20, for example anxiety, depression, social isolation, stress? Professional support may come from a counsellor, a psychologist, psychiatrist, or therapist.
    - Yes
    - No
    - Prefer not to say

22. If yes is selected:
    Approximately how much do you spend **per year** on professional support or guidance to help you manage anxiety, depression, social isolation, stress?
    - Free Text

23. Has providing care led to other impacts on your daily living?
    - Free Text

24. What would you say are the three most difficult aspects of providing care to someone living with a vision impairment?
    - Free Text

© 2024 Paudel N et al. *JAMA Ophthalmology.*
