# Supplementary material for: Economic Burden of Late-Stage Age-Related Macular Degeneration in Bulgaria, Germany, and the US
Source: JAMA Ophthalmol. 2024 Oct 31;142(12):1123–30. doi: 10.1001/jamaophthalmol.2024.4401 (PMC12316190; doi:10.1001/jamaophthalmol.2024.4401)
Supplement: Supplement 2. — Data sharing statement [file jamaophthalmol-e244401-s002.pdf]

Data Sharing Statement

Paudel. Economic Burden of Late-Stage Age-Related Macular Degeneration in Bulgaria, Germany, and the US. *JAMA Ophthalmol*. Published October 31, 2024. doi:10.1001/jamaophthalmol.2024.4401

Data
Data available: No

Additional Information
Explanation for why data not available: The survey data related to the study can be available from the corresponding author upon reasonable request.
